# Modeling the impact of calorie‐reduction interventions on population prevalence and inequalities in childhood obesity in the Southampton Women's Survey

**DOI:** 10.1002/osp4.520

**Published:** 2021-05-17

**Authors:** Simon J. Russell, Steven Hope, Helen Croker, Sarah Crozier, Jessica Packer, Hazel Inskip, Russell M. Viner

**Affiliations:** ^1^ Obesity Policy Research Unit Population, Policy and Practice Great Ormond Street Institute of Child Health University College London London UK; ^2^ MRC Lifecourse Epidemiology Unit Medicine University of Southampton Southampton UK; ^3^ NIHR Applied Research Collaboration Wessex Southampton Science Park Innovation Centre Southampton UK; ^4^ NIHR Southampton Biomedical Research Centre University of Southampton and University Hospital Southampton NHS Foundation Trust Southampton UK

**Keywords:** causal modeling, child and adolescent health, dietary interventions, health inequalities, obesity, Southampton Women's Survey

## Abstract

**Background:**

In the United Kingdom, rates of childhood obesity are high and inequalities in obesity have widened in recent years. Children with obesity face heightened risks of living with obesity as adults and suffering from associated morbidities. Addressing population prevalence and inequalities in childhood obesity is a key priority for public health policymakers in the United Kingdom and elsewhere. Where randomized controlled trials are not possible, potential policy actions can be simulated using causal modeling techniques.

**Objectives:**

Using data from the Southampton Women's Survey (SWS), a cohort with high quality dietary and lifestyle data, the potential impact of policy‐relevant calorie‐reduction interventions on population prevalence and inequalities of childhood obesity was investigated.

**Methods:**

Predicted probabilities of obesity (using UK90 cut‐offs) at age 6–7 years were estimated from logistic marginal structural models adjusting for observed calorie consumption at age 3 years (using food diaries) and confounding. A series of policy‐relevant intervention scenarios were modeled to simulate reductions in energy intake (differing in effectiveness, the targeting mechanisms, and level of uptake).

**Results:**

At age 6–7 years, 8.3% of children were living with obesity, after accounting for observed energy intake and confounding. A universal intervention to lower median energy intake to the estimated average requirement (a 13% decrease), with an uptake of 75%, reduced obesity prevalence by 1% but relative and absolute inequalities remained broadly unchanged.

**Conclusions:**

Simulated interventions substantially reduced population prevalence of obesity, which may be useful in informing policymakers.

## 1 INTRODUCTION

In England, rates of childhood overweight and obesity remain high and, in 2019/2020, 23.0% of reception‐age school children (4–5 years) were living with overweight or obesity.^1^ Children with obesity face heightened risks of living with obesity as adolescents and adults^2^ and suffering from associated morbidities.^3^ Socioeconomic disadvantage is a key determinant of childhood obesity,^4^ ^,^ ^5^ and inequalities in obesity have widened in the United Kingdom in recent years.^1^

Addressing population prevalence and inequalities in childhood obesity is a key priority for public health policymakers in the United Kingdom and elsewhere and has been undertaken in various ways. Policy goals to address the “top down” determinants of obesity, including food systems and industry, have been introduced. For example, the soft drink industry levy in the United Kingdom was a fiscal measure introduced as part of a challenge to all sectors of the food industry in 2015 to reduce sugar by 20% in food categories that primarily contribute to children’s sugar intake.^6^ Policy goals also relate to enabling healthier lifestyles with “bottom up” approaches, often focusing on lifestyle interventions that aim to reduce prevalence in obesity through behavior change. Typically, prevention and treatment interventions aim to address energy balance by reducing intake, improving diet quality or by increasing physical activity.

Trials of prevention and treatment interventions in children with obesity are often small in scale. Conducting trials at a population level is often not feasible, given the large‐scale, or timely, given the required recruitment and follow up times, for the investigation of interventions to inform national policy goals. Scaling up small‐scale interventions that have shown effectiveness to general populations can also be problematic.^7^ While it is reasonable to extrapolate effect sizes from trials to population models, scaled up prevention interventions are likely to have lower effects than the preceding efficacy trials. This is often due to interventions being trimmed back in order that they are responsive to implementation in real world contexts; additionally, the efficacy of large‐scale interventions are more difficult to assess.^8^ Treatment interventions that have established trial effectiveness in reducing BMI are also likely to be less effective when scaled up.^8^ Despite the potential difficulties, there is a policy need to produce evidence that can inform the development of calorie‐reduction policy goals.

This work builds on previous research using causal modeling techniques in the Avon Longitudinal Study of Parents and Children (ALSPAC) that applied calorie‐reduction interventions to children aged 7 years and measured the potential impact on population prevalence and inequalities in childhood obesity at 11 years.^9^ This work sought to replicate previous analysis among younger children and in a second cohort, the Southampton Women's Survey (SWS), a longitudinal cohort of Southampton women and their children. Scenarios were modeled to represent interventions (reductions in energy intake at age 3 years), which varied in effectiveness, uptake and targeting, with estimated impacts on prevalence and inequalities in obesity at age 6–7 years. Given that randomized controlled trials are not always possible for general population interventions, these analyses may be useful to policy makers in demonstrating the potential impact of calorie‐reduction interventions on population prevalence of childhood obesity.

## 2 METHODS

### 2.1 Data sources

The SWS was used, a large prospective cohort study of mothers and children that recruited 12,583 non‐pregnant Southampton women aged 20 to 34 years from the general population between 1998 and 2002. Between 1998 and 2007, 3158 participants became pregnant and were invited to take part in the pregnancy phase of the survey. The survey followed up children with home visits at 6 months, 1 year, 2 years, and 3 years; further samples of children were seen at 4 years and 6–7 years. Further details of the cohort profile have been previously published.^10^ Ethical approval was granted by the Southampton and South West Hampshire Local Research Ethics Committee.

### 2.2 Analytic sample

The whole SWS sample comprised 3158 mothers; there were complete data on maternal education (exposure) for 3149 (99.7%) mothers, and on BMI (outcome) for 2007 children at age 6–7 years (63.6%). Total daily calories were available from 2‐day food diaries recorded for 893 children at age 3 years (28.3%). There were also 247 records with missing data for confounding variables, resulting in a complete case sample of 646 (20.5%) children. The analytic sample was derived by restricting the total sample to children with complete data on maternal education (exposure) and BMI at age 6–7 years (outcome) (*n* = 2001). Multiple imputation by chained equations (assuming data were missing at random) was used to deal with attrition and missing data for the mediator and confounding variables (Supplement 1).^11^ While the sample of participants that completed food diaries was small, the analytic sample (using imputed data) was found to be similar to the whole sample (Table 2).

### 2.3 Measures

#### 2.3.1 Exposure (maternal education)—pre pregnancy

Given the relationship between socioeconomic disadvantage and childhood obesity, highest maternal education (recorded pre‐pregnancy) was conceptualized as an upstream marker of inequalities and used as a household measure of socioeconomic position. Due to small numbers, the original six categories were collapsed into three groups for analyses: high level of education (“Degree”), middle level of education (“O level,” “A level,” or “Higher National Diploma”), and low level of education (“None” or “CSE”).

#### 2.3.2 Outcome (obesity) at 6–7 years

Weight and height were objectively measured by research nurses and used to calculate BMI z‐scores using the UK90 reference data.^12^ Cut offs for epidemiological application^13^ were applied for overweight (>85th percentile) and obesity (>95th percentile).

#### 2.3.3 Mediator (total daily calorie consumption) at 3 years

Calorie‐intake was considered a downstream mediator of the relationship between disadvantage and obesity. In SWS, dietary data were collected at all data collection waves using food frequency questionnaires, supplemented at age 3 years with 2‐day prospective food diaries. For these analyses, food diaries were used to calculate daily calorie intake as they were judged to represent dietary intake more accurately than food frequency questionnaires (diaries require less recall and recorded actual intake for specific days).^14^ Following an interview, mothers were invited by a research nurse to complete diaries on behalf of the child, recording in their own words all food and drink consumed by the child from midnight the day following the interview until midnight 2 days later.^15^ Food and drink items were described by number, measure (e.g., tablespoon), size and weight; cooking methods and dietary supplements were also recorded.^15^ Completed diaries were returned by post and were checked for completeness by a member of the research team. Mothers/caregivers were contacted for clarification if there were missing or illegible data. Nutrient intakes from diet diaries were calculated by multiplying portion weights and nutrient contents of each food; food composition was based on McCance and Widdowson 5th Edition^16^ and supplementary volumes. Where required, food recipes and composition of dietary supplements were provided by manufacturers.

#### 2.3.4 Estimated average requirements

To inform interventions, estimated average requirements (EAR) were used to indicate food energy needs for children aged 3 years.^17^ EARs are derived by multiplying the basal metabolic rate (BMR) with physical activity level (PAL), after adjusting for growth and development.^17^ The EAR for children aged 3 years is estimated to be 1171.0 kcals for boys and 1076.0 kcals for girls. Median intake in calories in this sample at age 3 years (taken from food diaries) was estimated to be 1311.5 for boys and 1273.5 for girls. A quantitative estimate of effective and healthy long‐term weight loss was adapted from recommendations for adults.^18^

### 2.4 Confounding

**FIGURE 1:**
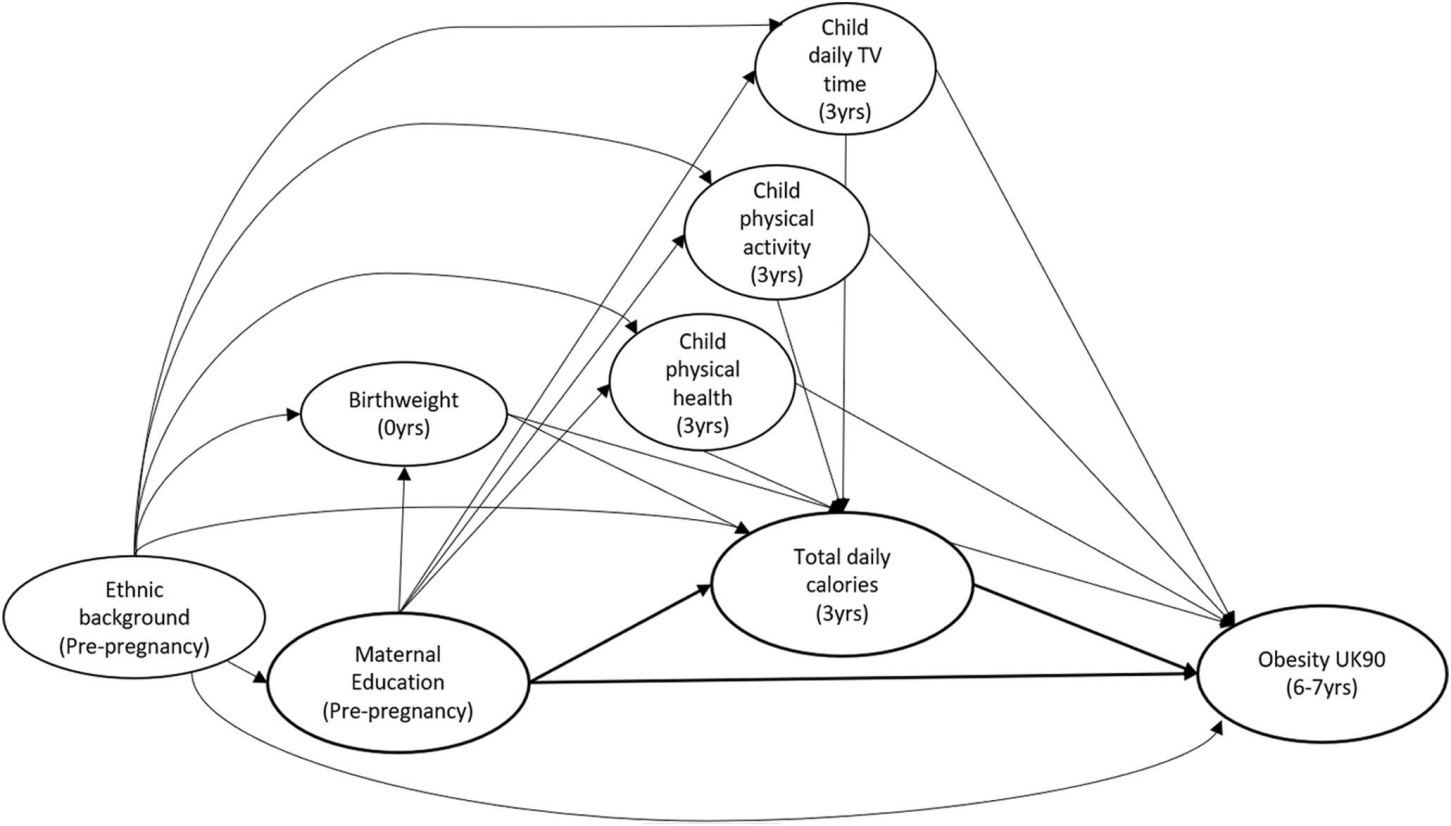
Directed acyclic graph showing theoretical associations between exposure (maternal education at pre‐pregnancy), mediator (estimated energy intake in calories at 3 years), and outcome (BMI z‐scores at 6–7 years)

Confounding factors were identified using a directed acyclic graph (Figure 1), which demonstrates the associations between disadvantage (exposure) to childhood obesity (outcome) with energy intake (mediator), accounting for potential baseline and intermediate confounding.

#### 2.4.1 Baseline confounding

Child ethnicity, categorized as “white” and “non‐white.”

#### 2.4.2 Intermediate confounding

Standardized birthweight was obtained from obstetric data, categorized as low (less than 1 SD below the mean), mid (–1 SD to +1 SD), or high (greater than 1 SD above the mean). All other variables were from mother‐report at 3 years: general health of the child (categorized good health vs. fair/bad health); moderate daily physical activity of the child (categorized as low [0–4 h per day], mid [5–8 h per day], and high [9–12 h per day]); and daily TV time of the child, categorized as low (less than 1 h per day), mid (1.5–2.5 h per day), and high (more than 3 h per day).

#### 2.4.3 Targeted and indicated variables

Interventions were either universal (for all children), targeted based on risk of obesity, or indicated based on prior obesity. Variables used in targeted and indicated scenarios were recorded at approximately the same time as daily calorie intake.

#### 2.4.4 Deprivation

Index of Multiple Deprivation (IMD) recorded in 2004 was used as a targeting variable for an intervention as children from deprived areas were determined to be at heightened risk of obesity.^4^, ^5^ A geographically targeted intervention could indirectly address inequalities in obesity.

#### 2.4.5 High energy intake

High estimated energy intake (boys and girls with estimated intake greater than the EAR) was used for an intervention to target children at greater individual risk of obesity.

#### 2.4.6 Prior obesity

Prior weight status (BMI at age 3 years) was used as an indicator variable for children at heightened risk of obesity.^2^ Obesity at age 3 years was indicated using z‐scores from UK90 reference data^12^ and cut‐offs for epidemiological application.^13^

### 2.5 Data analysis

Regression analyses were conducted to estimate relationships between maternal education (the exposure at baseline), average daily calorie intake (the mediator at age 3 years), z‐scores for BMI (the outcome at age 6–7 years), and confounding variables. The distribution of energy intake was skewed and therefore median daily calories were shown in descriptive statistics. Obesity prevalence at 6–7 years was reported by maternal education group with relative and absolute inequalities.

Logistic regression within a marginal structural modeling (MSM) framework^9^ ^,^ ^19^ ^,^ ^20^ was used to model the association between maternal education and BMI at 6–7 years. In the unadjusted model, predicted probabilities with 95% confidence intervals were used to estimate childhood obesity prevalence overall and by maternal education group. For the adjusted model, average daily calories was included as a continuous variable and baseline and intermediate confounding were accounted for using inverse probability weights (IPWs; truncated between 1% and 99%). Probabilities from the adjusted model were used to derive the control direct effect (CDE), which gives the estimated effect of maternal education on obesity when daily energy intake was fixed at the observed level.^21^ The CDE was the model against which effect size estimates from simulations were compared.

Relative inequalities are given as risk ratios (the ratio of fitted probabilities of obesity for the highest to the lowest maternal education groups) and absolute inequalities are given as risk differences (the difference between the fitted probabilities between the highest and lowest maternal educational groups), with maternal educational level entered as a continuous term. Stata SE 15.1 was used to perform all analyses.^22^

#### 2.5.1 Simulations

To simulate policy actions or interventions that reduced energy intake, adjustments (reductions) were made to the mediator (daily calorie intake). Simulation scenarios represent potential interventions according to: effectiveness (magnitude of calorie reduction); targeting or indicated (high deprivation, prior weight status, or reported calorie intake); and, uptake of intervention among eligible children (Table 1). Variability was reflected in each reduction.

**TABLE 1:** Simulated intervention scenarios

Scenarios	Calorie reduction	Target	Uptake
1. Universal intervention to meet estimated average requirements (EAR)	−13.0% (−10.7% boys, −15.5% girls)	All children	75%
2. Targeted intensive intervention for children from highly deprived areas	−21.3%	High relative deprivation (33.8%)	75%
3. Indicated intensive intervention for children with prior obesity	−21.3%	Children living with obesity at age 3 years (6.7%)	100%
4. Targeted intervention for children consuming excess total daily calories	Variable	Boys consuming >1171 kcal per day (66.5%) and girls consuming >1076 kcal per day (78.0%) (72.1% overall)	100%

Scenario 1 modeled the impact of a universal intervention that reduced population intake of calories for children aged 3 years. All children were considered eligible and uptake of the intervention was set at 75%. The reduction applied was based on the difference between the observed median intake and the EAR. Sex‐specific reductions were applied with variation by creating a normal distribution around the adjusted level. To reduce median intake to the EAR, boys’ intake was reduced by 140.5 calories (a 10.7% decrease in observed intake) and girls’ by 197.5 (a 15.5% decrease). This equated to an overall reduction in calories in the sample population of 13.0%.

Scenario 2 modeled an intensive intervention with increased effectiveness (a greater reduction), targeted to children living in deprived areas, with uptake of eligible children set at 75%. Using IMD 2004, deprived areas were defined as 1 SD above the mean sample score. The effectiveness (reduction in calories) was greater in this simulated intervention as it was administered to children at heightened risk of obesity. The reduction was informed by EAR values for adults (sex and age groups combined),^17^ and the effectiveness was guided by a recommendation for healthy, long‐term weight loss in adults (reducing intake by 500 fewer calories per day).^18^ The intensive intervention equated to a 21.3% decrease in daily energy intake for the targeted group. The intensive intervention was not sex‐specific as it was adapted from generic weight loss guidance for male and female adults.

Scenario 3 modeled the intensive intervention described in Scenario 2 (a 21.3% reduction in daily intake) for children at heightened individual risk of obesity. This was indicated by past weight status; all children living with obesity at age 3 years (6.7%) were eligible and received this intervention.

Scenario 4 modeled a targeted intervention that limited intake in children reported to be consuming high daily calories. This intervention truncated the population distribution of energy intake. For the targeted children, intake was reduced to the level of the EAR so that every boy (1171 kcals) and girl (1076 kcals) in the analytic sample had an intake that was equal to or less than the EAR.

In scenarios 1 and 2, uptake was set at 75% since not all children in a population would be expected to comply with an intervention. In scenarios 3 and 4, uptake was set at 100% and total engagement with the interventions by target populations was assumed.

For all simulations, a lower bound was set at 2 SD below mean intake reported from food diaries in the analytic sample, guided by the lower nutrient intake bound,^23^ and was set to prevent energy intake being reduced among children with low calorie intake.

### 2.6 Sensitivity analyses

Three sensitivity analyses were carried out. First, models were estimated in a complete case sample (*n* = 646). Second, analyses were repeated using maternal social class as an alternative indicator of socioeconomic circumstances. Third, S1 was run with higher and lower levels of intervention uptake (100% and 50%) reflecting compliance with a policy measure or intervention.

## 3 RESULTS

### 3.1 Descriptives

Of mothers in the analytic sample, 12.5% were from the lowest education group (Table 2). At age 3 years, median energy intake was 1292.6 kcals overall and 1311.5 kcals for boys and 1273.5 kcals for girls, which equated to an excess of 140.5 and 197.5 calories respectively compared to the EAR. The percentage of children with reported energy intake equal to or less than the EAR was 33.5% for boys and 22.0% for girls. Obesity prevalence at age 6–7 years was 8.3%.

**TABLE 2:** Descriptive statistics of Southampton Women's Survey across analytical samples

	Whole sample (*n* = 3158)	Complete case (*n* = 646)	Imputed sample (*m* = 50) *n* = 2001
Sex			
Male	(1633) 51.8%	(334) 51.7%	51.3%
Female	(1520) 48.2%	(312) 48.3%	48.7%
Missing	(5)	‐	‐
Exposure			
Highest maternal education			
Low	(394) 12.5%	(66) 10.2%	9.4%
Mid	(2062) 65.5%	(399) 61.8%	66.4%
High	(693) 22.0%	(181) 28.0%	24.2%
Missing	(9)	‐	‐
Baseline confounding (0 years)			
Ethnicity			
White	(3016) 95.5%	(625) 96.7%	96.1%
Non‐white	(139) 4.4%	(21) 3.3%	3.9%
Missing	(3)	‐	‐
Mediator			
Total daily calories			
Median kcal (SE)	1288.3 (9.3)	1281.1 (11.0)	1292.6 (12.2)
Missing	(2265)	‐	‐
Intermediate confounding			
Birthweight			
Low	(380) 12.2%	(68) 10.5%	13.1%
Mid	(2314) 74.2%	(498) 77.1%	72.8%
High	(425) 13.6%	(80) 12.4%	14.1%
Missing	(39)	‐	‐
Child physical health			
Good health	(2470) 95.1%	(625) 96.7%	95.6%
Fair/bad health	(128) 4.9%	(21) 3.3%	4.4%
Missing	(560)	‐	‐
Moderate activity			
Low (≤4 h per day)	(646) 25.4%	(150) 23.2%	24.3%
Mid (5–8 h per day)	(1696) 66.7%	(442) 68.4%	68.0%
High (≥9 h per day)	(201) 7.9%	(54) 8.4%	7.7%
Missing	(615)	‐	‐
Daily TV time			
Low (≤1 h per day)	(520) 20.5%	(135) 20.9%	20.9%
Mid (1.5–2.5 h per day)	(1764) 69.5%	(428) 66.3%	69.0%
High (>2.5 h per day)	(253) 10.0%	(83) 12.9%	10.1%
Missing	(621)	‐	‐
Outcome			
BMI status (6–7 years)			
Without overweight/obese	(1591) 79.3%	(527) 81.6%	81.8%
Overweight (85^th^–95^th^)	(206) 10.3%	(66) 10.2%	9.8%
Obese (>95^th^ centile)	(210) 10.5%	(53) 8.2%	8.3%
Missing	(1151)	‐	‐
Targeting/indicating variables for interventions			
BMI status (3 years)			
Not overweight/obese	(2079) 82.9%	(537) 84.8%	83.1%
Overweight (85^th^–95^th^)	(265) 10.6%	(57) 9.0%	10.4%
Obese (>95^th^)	(164) 6.5%	(39) 6.2%	6.7%
Missing	(650)	‐	‐
IMD			
Quintile 1—least deprived	(633) 20.0%	(151) 23.4%	22.5%
Quintile 2	(547) 17.3%	(109) 16.9%	17.8%
Quintile 3	(773) 24.5%	(174) 26.9%	25.8%
Quintile 4	(746) 23.6%	(141) 21.8%	21.2%
Quintile 5—most deprived	(459) 14.5%	(71) 11.0%	12.6%

Maternal education was associated with childhood obesity at age 6–7 years but in these analyses, the marker for disadvantage was a property of the mother, not the child. Children with mothers in the low educational group were 2.2 times more likely to be living with obesity compared to those with mothers in the highest educational group. Daily calorie intake reported from food diaries at age 3 years was positively associated with higher BMI z‐scores and obesity status at age 6–7 years (*p* = 0.01). Median calorie consumption was greatest in children with mothers in the highest educational group, but there was no apparent relationship between maternal educational and energy intake (Table S2). Potential baseline and intermediate confounders were associated with maternal education (exposure) and childhood obesity at age 6–7 years (outcome) but were not associated with estimated energy intake (mediator). Relationships between confounding variables and exposure, mediator, and outcome variables are shown in Table S3.

### 3.2 Simulated interventions

In the unadjusted model, population prevalence of obesity for children aged 6–7 years was 8.4%; 10.1% in the lowest education group and 4.8% in the highest. The estimated population prevalence of obesity for children aged 6–7 years in the CDE model was 8.3% (after adjustment for children’s reported energy intake at age 3 years and confounding) with inequalities observed (Table 3). Children in the lowest education group were found to be 2.1 times more likely to be living with obesity when compared with the highest. Prevalences in the unadjusted and CDE models were similar, suggesting that reported energy intake did not strongly mediate the relationship between maternal education and childhood obesity.

**TABLE 3:** Prevalence of obesity at age 6–7 years by maternal educational level with risk ratios and differences for relative and absolute inequalities, for intervention scenarios 1‐4

Scenario	Consuming ≤EAR (boys/girls)	Prevalence of obesity at 6–7 years (≥95^th^ centile)					
		Overall (% change vs. CDE)	Highest maternal education level			Inequalities in obesity^a^	
			Low (% change vs. CDE)	Mid (% change vs. CDE)	High (% change vs. CDE)	Risk ratio^b^ (CIs)	Risk difference^b^ (CIs)
Unadjusted model							
	33.5%/22.0%	8.4%	10.1%	9.4%	4.8%	2.2 (1.1–3.3)	6.9 (2.1–11.7)
Control direct effect (CDE)^c^							
	33.5%/22.0%	8.3%	9.7%	9.4%	4.7%	2.1 (1.1–3.2)	6.6 (2.2–11.0)
Simulation 1: Universal intervention to reduce average intake down in line with estimated average requirements (EAR) (−13.0% overall), 75% uptake							
	51.7%/37.7%	7.3% (−11.9%)	8.5% (−11.5%)	8.2% (−12.0%)	4.2% (−12.1%)	2.2 (1.1–3.2)	6.0 (2.0–10.0)
Simulation 2: Targeted intensive intervention (−21.3%) for children from more deprived areas, 75% uptake							
	44.0%/31.5%	7.7% (−6.4%)	8.7% (−9.7%)	8.8% (−6.4%)	4.5% (−4.4%)	2.0 (1.0–3.0)	5.6 (1.5–9.7)
Simulation 3: Indicated weight loss intervention (−21.3%) for children with obesity (6.7%), 100% uptake							
	36.1%/23.9%	8.1% (−1.9%)	9.4% (−2.4%)	9.2% (−2.1%)	4.7% (−1.3%)	2.1 (1.1–3.1)	6.4 (2.1–10.7)
Simulation 4: Targeted calorie‐reduction simulation for children consuming excess total daily calories (72.1%) to limit intake to EAR, 100% uptake							
	100%/100%	6.4% (−22.5%)	7.5% (−22.1%)	7.3% (−22.4%)	3.6% (−23.1%)	2.2 (1.1–3.3)	5.3 (1.5–9.0)

^a^ Relative and absolute inequalities were estimated using a continuous linear term for highest maternal education level.

^b^ Risk ratios and differences are likelihoods calculated with reference to non‐obese group (<95th centile of zBMI at age 6–7 years).

^c^ The effect of maternal educational level on obesity prevalence at age 6–7 years, adjusted for baseline and time‐varying confounding with mediation of total daily calories held at observed level.

Scenario 1 (a universal calorie reduction of 13%): Overall obesity prevalence reduced by 1.0%, to 7.3%. Relative inequalities remained unchanged and absolute inequalities slightly narrowed, reflecting the decrease in the population prevalence. The proportion of children with calorie intake equal to or less than the EAR increased from 33.5% to 51.7% for boys and from 22.0% to 37.7% for girls in this scenario.

Scenario 2 (an intensive 21.3% reduction in energy intake, targeted to children living in deprived areas): Obesity prevalence reduced and absolute inequalities remained unchanged.

Scenario 3 (an intensive 21.3% reduction in energy intake targeted to children with obesity at age 3 years): Estimated prevalence and inequalities in obesity were broadly unchanged.

Scenario 4 (an intervention targeted at children with daily intake exceeding the EAR): Reductions were applied to children consuming in excess of the EAR so that the maximum calorie intake was limited to the average requirement for children aged 6–7 years. Obesity prevalence was reduced, relative inequalities were unaffected, while absolute inequalities were reduced, likely driven by general trends in prevalence.

### 3.3 Sensitivity analysis

Results from models using social class as an alternate exposure were similar to those reported for maternal education (Table S4). Simulation 1 was repeated with 100% and 50% uptake. As expected, the pattern of results was similar but with a greater reduction in obesity prevalence for 100% uptake and a smaller reduction in obesity prevalence for 50% uptake (Table S5).

## 4 DISCUSSION

Childhood obesity is common in reception age children (4–5 years) and there is a social gradient with the most disadvantaged being the most likely to be obese. In these analyses, obesity prevalence was 8.3% for children age 6–7 years and was predicted by maternal education. Models simulating the effects of reducing energy intake at age 3 years reduced obesity prevalence and reductions were proportional to the effectiveness of the hypothetical intervention. In simulations of universal interventions, decreases in obesity prevalence were greatest in the lowest maternal education group but the percentage change (proportional to the prevalence in the CDE model) was greatest in the highest education group. In this and prior work, estimated calorie intake was not associated with indicators of disadvantage, and inequalities remained broadly unchanged following simulations, likely due to the lack of social patterning of energy intake. Prevalences in the unadjusted and CDE models were similar, meaning that energy intake showed little mediating effect on the exposure‐outcome pathway. These findings highlight the strength and complexity of the association between disadvantage and obesity.

Improving dietary behavior is a key focus of policy makers in the United Kingdom^23^ and elsewhere, and reducing the amount of energy consumed has been identified as an important step in tackling childhood obesity. Extrapolation of trial evidence can be problematic given that scaled up prevention and treatment interventions have been shown to be less effective than when carried out in treatment trials.^8^ ^,^ ^24^ Given that the potential impact of policy action to limit dietary intake in children in unknown, simulating potential calorie‐reduction interventions can provide useful insight to policy makers.

These analyses built upon previous work^9^ and further established MSMs to be potentially useful in estimating the effects of hypothetical interventions that reduce energy intake at a population level. A simulation approach was applied, using the SWS cohort, to estimate the potential impacts of calorie reduction interventions on population prevalence and inequalities in childhood obesity. These analyses were carried out on younger age groups; prior work considered energy intake at age 7 years and impacts on obesity at age 11 years, whereas these analyses considered the mediator at age 3 years and the outcome at age 6–7 years. Children aged 3 years in the SWS cohort were found to be overeating relative to the EAR and proportionally more than children aged 7 years in the ALSPAC cohort. Energy‐reduction interventions were found to reduce population prevalence of obesity in children and effect sizes were greater than in the ALSPAC cohort (for older children) both in terms of absolute reductions and change in prevalence relative to the CDE.

The findings from these analyses are likely to be relevant to contemporary populations since rates of obesity (and assumed intake) have remained relatively stable for reception‐age children since the data collection periods (pre‐2007 in the SWS).^1^ However, for older children, rates of obesity have increased; therefore, higher levels of intake that are likely among contemporary populations would require greater reductions in calorie consumption in order to meet the average requirements. That greater effect sizes were observed for hypothetical interventions at age 3 years compared with age 7 years in the ALSPAC cohort, imply that calorie reduction interventions may be more effective when administered earlier in childhood. However, it is not possible to disentangle the varying effects of age, period of data collection or the cohort studies themselves.

Population interventions to reduce calorie intake in early childhood would be likely to reduce prevalence of childhood obesity. This research used high quality longitudinal data from a regional cohort, including objectively recorded BMI, food diaries to generate average daily energy intake, and a number of potential confounding factors. EARs were used to guide adjustments to calorie intake (the effectiveness of hypothetical interventions). Given the high and increasing rates of obesity in the United Kingdom, these estimates are based on an approach that referenced body weights consistent with good long term health.^17^ The 95^th^ centile of BMI was used as the cut‐off for the obesity outcome, as this cut‐off has policy relevance and comparability across data sets.

Uptake was set at 75% for the universal intervention and the targeted intervention for children in deprived areas, and 100% for children living with obesity and with high levels of reported intake. It is acknowledged that modeled uptakes are high and may not be achievable in the real world. In sensitivity analyses, a lower level of uptake was modeled with a resulting pattern of results that was similar to those reported in the main analyses. Scenarios were modeled to be relevant to the real world and were targeted or indicated based on deprivation, prior weight status or eating behavior in order to provide insight as to the potential impacts on sub‐groups using different indicators of risk.

There are limitations with the regional dataset; the city of Southampton is generally more deprived and less ethnically diverse than England and Wales nationally.^10^ A slightly lower proportion of SWS mothers had university degrees compared to the national average during the same time period.^25^ The cohort of children were born pre‐2007 and may not reflect the experiences or behaviors of contemporary children but the overall associations are unlikely to be affected.

Estimated intake was based on two‐day mother‐report food diaries, which are generally thought to be valid^26^ ^,^ ^27^ but may contain a level of report bias.^28^ Furthermore, food diaries were only returned by a minority of mothers, who tended to have higher levels of education than those who did not, and to have children who were less likely to be living with overweight or obesity. These limitations would not have undermined the estimated reductions in prevalence or the relationships within the model, suggesting that the underlying mechanisms linking the exposure, mediator and outcome are likely to be generalizable. There may be potential caveats with the hypothesized causal pathway and limitations of quantifying this structure using survey data. There may be remaining unmeasured confounding in the conceptualized model, meaning causation cannot necessarily be assumed from these analyses.^29^

## 5 CONCLUSIONS

Calorie reduction interventions are a promising area for policy focus and may be helpful in tackling high rates of childhood obesity. This work supports the findings from previous studies and suggests that policy actions or interventions to reduce energy intake would be likely to lower the prevalence of childhood obesity. This work also implies that calorie reduction interventions may be more effective when administered earlier in childhood. While universal interventions did not significantly narrow relative inequalities, targeting children from highly deprived areas was effective in substantially reducing obesity prevalence among the lowest educational group. Given the stark and increasing inequalities in childhood obesity, policy actions and interventions that tackle inequalities should remain top of the public health agenda.

## CONFLICT OF INTERESTS

The authors have no competing interests to declare.

## AUTHOR CONTRIBUTIONS

Simon J. Russell, Russell M. Viner, and Steven Hope conceived the idea and designed the work; Simon J. Russell conducted the analyses; Simon J. Russell, Steven Hope, Helen Croker, Jessica Packer, and Russell M. Viner contributed to interpretation of results and writing of the paper. Hazel Inskip runs the SWS, provided the data, and contributed to discussions of the manuscript. Sarah Crozier curates the SWS data and contributed to discussions of the manuscript. All authors approved the submitted version and agree to be personally accountable for their respective contributions.

## Supporting information

Supplementary Material
