# Supplementary material for: Modeling the impact of calorie‐reduction interventions on population prevalence and inequalities in childhood obesity in the Southampton Women's Survey
Source: Obes Sci Pract. 2021 May 17;7(5):545–54. doi: 10.1002/osp4.520 (PMC8488449; doi:10.1002/osp4.520)
Supplement: Supplementary file 1 — Supplementary Material [file OSP4-7-545-s001.docx]

## Supplementary materials

**Supplement 1.**

Further details of multiple imputation

Maternal age, maternal education, and maternal social class were registered as regular variables, in addition to IMD scores in 2004, which was used to inform uptake scenarios, and BMI at age 3 years, which was used for targeted interventions. Mother-reported marital status, collected at baseline, and mother age at time of delivery, were used as auxiliary variables for the multiple imputation. Both had complete or near complete observations and were significant in predicting missingness for the mediator and confounding variables.

Data were imputed using ordered logistic regression for maternal social class and child general health; predictive mean matching (to one of ten neighbouring data points) for TV watching per day; and linear regression for zBMI at 3 years, birthweight, physical activity per day and total daily calories. Augmented regression was used to overcome the presence of empty cells.

Imputed datasets were consistent with the complete case sample in terms of frequencies and summary statistics.

**Table S2.** Relationship between maternal educational and median total daily calories and obesity (n=2,001)

| **Maternal education** | **Median total daily calories (SE)** | **% obese 6-7 years** |
| --- | --- | --- |
| Low (None, CSE) | 1281.5 (35.6) | 10.0% |
| Mid (O level, A level, HND) | 1291.0 (15.0) | 9.4% |
| High (degree) | 1302.1 (21.0) | 4.8% |
| p-value | p=0.851 | p<0.001 |

**Table S3.** Relationship between baseline and Intermediate confounding and exposure, mediator and outcome variables (n=2,001)

|  | **Maternal education**  **% in lowest level** | **Total daily calories**  **3 years**  **Median (SE)** | **BMI**  **6-7 years**  **% obese** |
| --- | --- | --- | --- |
| Ethnicity |  |  |  |
| White | 9.5% | 1294.7 (12.4) | 8.2% |
| Non-white | 7.7% | 1234.9 (56.1) | 11.5% |
| p-value | <0.001 | p=0.288 | <0.001 |
|  |  |  |  |
| Birthweight |  |  |  |
| Low | 11.4% | 1263.0 (29.8) | 6.2% |
| Mid | 9.5% | 1294.5 (13.6) | 7.4% |
| High | 7.5% | 1317.9 (31.2) | 15.2% |
| p-value | <0.001 | p=0.416 | <0.001 |
|  |  |  |  |
| Child physical health |  |  |  |
| Good health | 9.3% | 1297.1 (12.6) | 8.1% |
| Fair/bad health | 13.8% | 1155.9 (75.2) | 12.7% |
| p-value | <0.001 | p=0.064 | <0.001 |
|  |  |  |  |
| Moderate physical activity per day |  |  |  |
| Low (<=4 hours per day) | 12.5% | 1288.2 (22.2) | 10.1% |
| Mid (5-8 hours per day) | 8.5% | 1296.3 (15.3) | 7.5% |
| High (>=9 hours per day) | 8.5% | 1282.2 (39.2) | 10.6% |
| p-value | <0.001 | p=0.909 | <0.001 |
|  |  |  |  |
| Daily TV time |  |  |  |
| Low (<=1 hour per day) | 6.2% | 1283.6 (23.1) | 7.7% |
| Mid (1.5-2.5 hours per day) | 9.2% | 1295.4 (14.4) | 8.1% |
| High (>2.5 hours per day) | 17.9% | 1303.2 (33.9) | 11.3% |
| p-value | <0.001 | p=0.863 | <0.001 |

P-values obtained from multinomial (maternal education), linear (total daily calories), and logistic regressions (obesity at 6-7 years)

**Table S4.** Sensitivity analyses: CDE and simulation 1 by an alternate exposure, maternal social class (n=2001).

| **Scenario** | **% consuming less <=EAR**  **(boys/girls)** | **Prevalence of obesity at 6-7 years (>=95^th^ centile)** | | | | **Inequalities in obesity^a^** | |
| --- | --- | --- | --- | --- | --- | --- | --- |
|  |  | **Overall**  **(% change**  **vs CDE)** | **Maternal social class** | | | **Risk ratio^b^**  **(CIs)** | **Risk difference^b^**  **(CIs)** |
|  |  |  | **Low**  **(% change**  **vs CDE)** | **Mid**  **(% change**  **vs CDE)** | **High**  **(% change**  **vs CDE)** |  |  |
| Control Direct Effect^c^ | | | | | | | |
|  | 33.5% / 22.0% | 8.2% | 8.6% | 8.5% | 7.8% | 1.1 (0.7 – 1.5) | 0.9 (-2.4 – 4.1) |
| Simulation 1: Universal intervention to meet kcal per day recommendation (-13.0% overall), 75% | | | | | | | |
|  | 51.5% / 37.5% | 7.2%  (-12.0%) | 7.6%  (-11.1%) | 7.5%  (-12.3%) | 6.8%  (-12.2%) | 1.1 (0.7 – 1.6) | 0.9 (-2.1 – 3.8) |

^a^ Relative and absolute inequalities were estimated using a continuous linear term for maternal social class.

^b^ Risk ratios and differences are likelihoods calculated with reference to non-obese group (<95th centile of zBMI at age 6-7 years).

^c^ The effect of maternal social class on obesity prevalence at age 6-7 years, adjusted for baseline and time-varying confounding with mediation of total daily calories held at observed level.

**Table S5.** Sensitivity analyses: CDE and simulation 1 (75% uptake) with 100%, and 50% uptake (n=2,001)

| **Scenario** | **% consuming less <=EAR**  **(boys/girls)** | **Prevalence of obesity at 6-7 years (>=95^th^ centile)** | | | | **Inequalities in obesity^a^** | |
| --- | --- | --- | --- | --- | --- | --- | --- |
|  |  | **Overall**  **(% change**  **vs CDE)** | **Maternal education** | | | **Risk ratio^b^**  **(CIs)** | **Risk difference^b^**  **(CIs)** |
|  |  |  | **Low**  **(% change**  **vs CDE)** | **Mid**  **(% change**  **vs CDE)** | **High**  **(% change**  **vs CDE)** |  |  |
| Control Direct Effect^c^ | | | | | | | |
|  | 33.5% / 22.0% | 8.3% | 9.7% | 9.4% | 4.7% | 2.1 (1.1 – 3.2) | 6.6 (2.2 – 11.0) |
| Simulation 1: Universal intervention to meet kcal per day recommendation (-13.0% overall), 75% uptake | | | | | | | |
|  | 51.7% / 37.7% | 7.3%  (-11.9%) | 8.5%  (-11.5%) | 8.2%  (-12.0%) | 4.2%  (-12.1%) | 2.2 (1.1 – 3.2) | 6.0 (2.0 – 10.0) |
| Simulation 1b: Universal intervention to meet kcal per day recommendation (-13.0% overall), 100% uptake | | | | | | | |
|  | 57.4% / 42.9% | 6.9%  (-15.9%) | 8.2%  (-15.2%) | 7.9%  (-15.8%) | 3.9%  (-16.5%) | 2.2 (1.1 – 3.2) | 5.7 (1.8 – 9.6) |
| Simulation 1c: Universal intervention to meet kcal per day recommendation (-13.0% overall), 50% uptake | | | | | | | |
|  | 45.9% / 32.3% | 7.6  (-7.9%) | 9.0%  (-7.2%) | 8.6%  (-7.9%) | 4.3%  (-8.3%) | 2.2 (1.1 – 3.2) | 6.2 (2.1 – 10.3) |

^a^ Relative and absolute inequalities were estimated using a continuous linear term for highest maternal education level.

^b^ Risk ratios and differences are likelihoods calculated with reference to non-obese group (<95th centile of zBMI at age 6-7 years).

^c^ The effect of maternal educational on obesity prevalence at age 6-7 years, adjusted for baseline and time-varying confounding with mediation of total daily calories held at observed level.
